# Vaccine side-effects and SARS-CoV-2 infection after vaccination in users of the COVID Symptom Study app in the UK: a prospective observational study

**DOI:** 10.1016/S1473-3099(21)00224-3

**Published:** 2021-07

**Authors:** Cristina Menni, Kerstin Klaser, Anna May, Lorenzo Polidori, Joan Capdevila, Panayiotis Louca, Carole H Sudre, Long H Nguyen, David A Drew, Jordi Merino, Christina Hu, Somesh Selvachandran, Michela Antonelli, Benjamin Murray, Liane S Canas, Erika Molteni, Mark S Graham, Marc Modat, Amit D Joshi, Massimo Mangino, Alexander Hammers, Anna L Goodman, Andrew T Chan, Jonathan Wolf, Claire J Steves, Ana M Valdes, Sebastien Ourselin, Tim D Spector

**Affiliations:** a Department of Twin Research & Genetic Epidemiology, King's College London, London, UK; b School of Biomedical Engineering & Imaging Sciences, King's College London, London, UK; c ZOE Global, London, UK; d Medical Research Council Unit for Lifelong Health and Ageing, Department of Population Science and Experimental Medicine, and Centre for Medical Image Computing, Department of Computer Science, University College London, London, UK; e Clinical & Translational Epidemiology Unit, Massachusetts General Hospital and Harvard Medical School, Boston, MA, USA; f Division of Gastroenterology, Department of Medicine, Massachusetts General Hospital and Harvard Medical School, Boston, MA, USA; g Diabetes Unit and Center for Genomic Medicine, Massachusetts General Hospital, Boston, MA, USA; h Department of Medicine, Harvard Medical School, Boston, MA, USA; i Program in Medical and Population Genetics, Broad Institute, Cambridge, MA, USA; j National Institute for Health Research (NIHR) Biomedical Research Centre at Guy's and St Thomas' Foundation Trust, London, UK; k Nottingham NIHR Biomedical Research Centre at the School of Medicine, University of Nottingham, Nottingham City Hospital, Nottingham, UK; l Department of Infection, Guy's and St Thomas' Foundation Trust, St Thomas Hospital, London, UK

## Abstract

**Background:**

The Pfizer-BioNTech (BNT162b2) and the Oxford-AstraZeneca (ChAdOx1 nCoV-19) COVID-19 vaccines have shown excellent safety and efficacy in phase 3 trials. We aimed to investigate the safety and effectiveness of these vaccines in a UK community setting.

**Methods:**

In this prospective observational study, we examined the proportion and probability of self-reported systemic and local side-effects within 8 days of vaccination in individuals using the COVID Symptom Study app who received one or two doses of the BNT162b2 vaccine or one dose of the ChAdOx1 nCoV-19 vaccine. We also compared infection rates in a subset of vaccinated individuals subsequently tested for SARS-CoV-2 with PCR or lateral flow tests with infection rates in unvaccinated controls. All analyses were adjusted by age (≤55 years *vs* >55 years), sex, health-care worker status (binary variable), obesity (BMI <30 kg/m^2^*vs* ≥30 kg/m^2^), and comorbidities (binary variable, with or without comorbidities).

**Findings:**

Between Dec 8, and March 10, 2021, 627 383 individuals reported being vaccinated with 655 590 doses: 282 103 received one dose of BNT162b2, of whom 28 207 received a second dose, and 345 280 received one dose of ChAdOx1 nCoV-19. Systemic side-effects were reported by 13·5% (38 155 of 282 103) of individuals after the first dose of BNT162b2, by 22·0% (6216 of 28 207) after the second dose of BNT162b2, and by 33·7% (116 473 of 345 280) after the first dose of ChAdOx1 nCoV-19. Local side-effects were reported by 71·9% (150 023 of 208 767) of individuals after the first dose of BNT162b2, by 68·5% (9025 of 13 179) after the second dose of BNT162b2, and by 58·7% (104 282 of 177 655) after the first dose of ChAdOx1 nCoV-19. Systemic side-effects were more common (1·6 times after the first dose of ChAdOx1 nCoV-19 and 2·9 times after the first dose of BNT162b2) among individuals with previous SARS-CoV-2 infection than among those without known past infection. Local effects were similarly higher in individuals previously infected than in those without known past infection (1·4 times after the first dose of ChAdOx1 nCoV-19 and 1·2 times after the first dose of BNT162b2). 3106 of 103 622 vaccinated individuals and 50 340 of 464 356 unvaccinated controls tested positive for SARS-CoV-2 infection. Significant reductions in infection risk were seen starting at 12 days after the first dose, reaching 60% (95% CI 49–68) for ChAdOx1 nCoV-19 and 69% (66–72) for BNT162b2 at 21–44 days and 72% (63–79) for BNT162b2 after 45–59 days.

**Interpretation:**

Systemic and local side-effects after BNT162b2 and ChAdOx1 nCoV-19 vaccination occur at frequencies lower than reported in phase 3 trials. Both vaccines decrease the risk of SARS-CoV-2 infection after 12 days.

**Funding:**

ZOE Global, National Institute for Health Research, Chronic Disease Research Foundation, National Institutes of Health, UK Medical Research Council, Wellcome Trust, UK Research and Innovation, American Gastroenterological Association.

## Introduction

The UK's Medicines and Healthcare products Regulatory Agency has given emergency use authorisation to three COVID-19 vaccines: the Pfizer-BioNTech mRNA vaccine (BNT162b2), the Oxford-AstraZeneca adenovirus-vectored vaccine (ChAdOx1 nCoV-19), and the Moderna mRNA vaccine (mRNA-1273). The first two vaccines have been rolled out across the UK since Dec 8, 2020, and Jan 4, 2021, respectively.^1^ In late December, 2020, based on advice from the Joint Committee on Vaccination and Immunisation,^2^ the UK Government decided to delay the administration of second doses.

Phase 3 trials reported the BNT162b2 vaccine to have an efficacy of 52% at 12 days after the first dose and of 95% after the second dose if administered 3–4 weeks apart in participants without previous SARS-CoV-2 infection.^3^ The effectiveness of this vaccine in reducing infection, severe disease, hospitalisation, and death with COVID-19 has been reported for the whole of Israel,^4^ with reanalysis of the data from Israel revealing it to be 90% effective 2 weeks after the first dose.^5^ The ChAdOx1 nCoV-19 trial found efficacy against symptomatic disease of 76% at 22–90 days after at least one standard dose.^6^, ^7^, ^8^

**Research in context**

**Evidence before this study**

We searched PubMed for articles published up to March 10, 2021, using the terms (“BNT162b2“ OR “mRNA Covid-19 Vaccine” OR “ChAdOx1 nCoV-19” OR “adenovirus-vectored Covid-19 vaccine”) AND (“effectiveness” OR “reinfection” OR “side-effects” OR “adverse effects” OR “reactogenicity” OR “phase IV”). We did not restrict our search by language or type of publication. Besides the original phase 1–3 trials, we found one published article and two preprints on data from Israel investigating the effectiveness of the Pfizer-BioNTech vaccine (BNT162b2), a preprint from the UK exploring the effectiveness of both the BNT162b2 and Oxford-AstraZeneca (ChAdOx1 nCoV-19) vaccines in individuals aged 70 years or older in the community, and a study that linked health records for all vaccinated people in Scotland to investigate COVID-19 hospitalisation and mortality after vaccination. No study investigated the prevalence of adverse effects of the vaccines and all studies reported both vaccines to be highly effective.

**Added value of this study**

In this large prospective observational study, we assessed adverse effects from the two COVID-19 vaccines in use in the UK at the time of writing (BNT162b2 and ChAdOx1 nCoV-19), as well as self-reported infection rates following one dose or two doses of BNT162b2 and one dose of ChAdOx1 nCoV-19. Reported side-effects were minor in severity and of short duration. Headache and fatigue were more common in women than in men, in people aged 55 years or younger than in people older than 55 years, and after the second than after the first dose. Individuals with known past SARS-CoV-2 infection were more likely to have adverse effects after the first dose than were those without known past infection. We found, in a community setting, that self-reported infection rates of those vaccinated with the BNT162b2 or ChAdOx1 nCoV-19 vaccines were significantly lower than infection rates in unvaccinated controls. Documented infection rates in our app after a single vaccine dose decreased by 58% (95% CI 54–62) at 12–20 days, 69% (66–72) at 21–44 days, and 72% (63–79) after 45–59 days following BNT162b2, and 39% (21–53) at 12–20 days and 60% (49–68) at 21–44 days following ChAdOx1 nCoV-19, compared with unvaccinated controls.

**Implications of all the available evidence**

Localised and systemic side-effects after vaccination are less common in a real-world community setting than reported in phase 3 trials, mostly minor in severity, and self-limiting. Our data will enable prediction of side-effects based on age, sex, and past COVID-19 status to help update guidance to health professionals to reassure the population about the safety of vaccines.

Surveillance in the general population is necessary at this stage during vaccination rollout.^9^ The OpenSAFELY collaboration^10^ implemented a framework for monitoring vaccine rollout and coverage in the UK via record linkage, Public Health England has reported early data on effectiveness in the older population prioritised for vaccination,^11^ and a prospective observational study investigated the association between the rollout of the first vaccine dose and COVID-19 hospital admission in Scotland.^12^ Other surveys, such as the SIREN study^13^ in health-care workers, will also link directly to health records to assess the real-life effectiveness and safety of the various phases of vaccine rollout. However, it takes time for such studies to come to fruition. Real-time data from app users can provide a faster view of the safety and effectiveness of COVID-19 vaccines.

The aim of this study was to investigate the adverse effects and infection rate of vaccinated people in a community (general population app users) scenario. We used data from 627 383 individuals who received the BNT162b2 or ChAdOx1 nCoV-19 vaccines between December, 2020, and March, 2021, and reported symptoms in real-time via the COVID Symptom Study app.^14^ A subset of individuals also reported receiving a PCR or lateral flow test.

## Methods

### Study design and participants

The COVID Symptom Study app^14^ was developed by health data company ZOE Global, with input from King's College London (London, UK), the Massachusetts General Hospital (Boston, MA, USA), Lund University (Lund, Sweden), and Uppsala University (Uppsala, Sweden). In the UK, it was launched in English on March 24, 2020. The app enables self-reported information related to SARS-CoV-2 infection to be captured. Individuals older than 18 years can sign up to the app without any restrictions. Individuals can also record information for dependents younger than 18 years. Use of the app was driven by referrals or word of mouth, the media, and eventually partnerships with charities and the Welsh and Scottish Governments.^15^ On first use, the app records self-reported location, age, and core health risk factors (body-mass index [BMI], smoking status, race or ethnicity, and presence of comorbidities, including cancer, diabetes, eczema, heart disease, lung disease, kidney disease, and hay fever), as well as employment status, such as being a health-care worker. With continued use, participants provide daily updates on symptoms experienced, SARS-CoV-2 test results (negative, pending, or positive), vaccines administered, and whether they are self-quarantining or seeking health care, including the level of intervention and related outcomes. Individuals without symptoms are encouraged to report through the app every day. Through direct updates to the app, new or modified questions are added in real time to capture data to test emerging hypotheses about COVID-19 symptoms and treatments. Versions 2.1.0–2.4.0 of the app were in use during the study period.

Participants were asked if they had been vaccinated for COVID-19 and, if so, to record the type of vaccine and date of administration. We included all UK app users reporting having received at least one dose of the two available vaccines (appendix p 2). Users reporting vaccination were then asked daily for the following 8 days whether they experienced adverse effects, including both systemic (whole body) and local effects. Systemic solicited side-effects included headache, fatigue, chills and shiver, diarrhoea, fever, arthralgia, myalgia, and nausea; solicited local side-effects included local pain, swelling, tenderness, redness, itch, warmth, and swollen armpit glands (appendix p 6). Users were also permitted to report no symptoms by leaving the box unchecked.

Ethical approval for use of the app for research purposes in the UK was obtained from King's College London Ethics Committee (review reference LRS-19/20-18210), and all users provided consent for non-commercial use of their data.

### Outcomes

Our primary outcome was the proportion of app users reporting adverse effects within 8 days after vaccination and the probability of having an adverse event. Our secondary outcome was infection rates in individuals after receiving a first dose of either the BNT162b2 or ChAdOx1 nCoV-19 vaccines. We did not collect information on why individuals were tested, so not all tested individuals were necessarily experiencing COVID-19-associated symptoms at testing, and some individuals might have been routinely tested while being asymptomatic.

### Statistical analysis

We used χ^2^ and Student's *t* tests to compare the demographic characteristics of individuals who received BNT162b2 versus those who received ChAdOx1 nCoV-19. We investigated the evolution of systemic and local adverse effects within 8 days from the vaccination date, computing the percentage of users experiencing side-effects after having received the vaccine. Vaccinated individuals who logged their systemic or local effects (or the absence of them) at least once within 8 days from the vaccination date were included in the adverse effects analysis (appendix p 2). We estimated the ratio of the daily number of users reporting at least one adverse effect (systemic or local) after vaccination to the total number of vaccinated users logging into the app that day. The occurrence of adverse effects was studied for both BNT162b2 doses and the first ChAdOx1 nCoV-19 dose.

We compared the probability of having adverse effects between the first and second BNT162b2 doses, the first BNT162b2 and the first ChAdOx1 nCoV-19 doses, and the second BNT162b2 and the first ChAdOx1 nCoV-19 doses. As different people received different vaccines, we used Pearl's back-door adjustment^16^ to account for differences within the populations. Backdoor adjustments are used when there are both causal and non-causal paths between predictors (eg, vaccine treatment [*V*]) and an outcome. Because of the observational (non-interventional) nature of the study, the non-causal paths between outcomes and predictors that might involve age, sex, BMI, or health status need to be adjusted statistically. Backdoor adjustment methods essentially condition on these variables, cutting out the non-causal (indirect) links between a predictor and an outcome:

P(R|do[V])=∑P(R|S,V)P(S)

where *R* is adverse effects, *S* is stratum, and *P*(*R | S,V*) is the probability of having adverse effects in a given stratum after receiving a vaccine.

We used the following strata: age (≤55 years *vs* >55 years), in line with stratification in the BNT162b2 and ChAdOx1 nCoV-19 phase 3 trials,^3^ sex, health-care worker status (binary variable), obesity (BMI <30 kg/m^2^ *vs* ≥30 kg/m^2^), and comorbidities (binary variable, with or without comorbidities). Adjusted odds ratios (ORs) were computed after Pearl's back-door adjustment was applied to the raw rates (see appendix p 15 for the formula). 95% CIs for ORs were obtained by bootstrapping 50 times on the vaccinated population.

Logistic regressions were used for each of the specified strata to investigate whether adverse effects varied across different participant groups, and in individuals who had previously reported a positive test for COVID-19 (PCR or lateral flow positive at least 6 months before vaccination, PCR or lateral flow positive within the 6 months before vaccination, and no previously detected infection).

Finally, in a subanalysis of vaccinated participants who reported having had the first dose of the BNT162b2 vaccine or one dose of the ChAdOx1 nCoV-19 vaccine and were then subsequently tested for SARS-CoV-2 infection, we investigated the change in infection rates after the first vaccine dose. We compared the outcomes of PCR or lateral flow tests in individuals who had been vaccinated with the first dose with those of unvaccinated individuals who reported having a COVID-19 test in the same week as a vaccinated app user. We computed the difference in days between when participants had the vaccine and when they were tested, and we used this metric to group users. If a person reported more than one PCR test or lateral flow result after being vaccinated, we selected the positive test, or if none were positive, the latest negative test. Since the BNT162b2 phase 3 trial showed a decrease in infections from 12 days after vaccination,^3^ we analysed infection rates at 0–4, 5–11, 12–20, 21–44, and 45–59 days after vaccination. For each of the vaccines and for different timepoints from the vaccination date, we used Poisson regressions to model the rates of positive tests in vaccinated individuals compared with those in the unvaccinated population, adjusting for the number of tests. This model allowed us to control for the number of follow-up days for each group. Moreover, as there was a two-times increase in daily incidence in England followed by a decrease of a similar proportion during the data collection period,^17^ we also included incidence as a covariate in the Poisson regression model, with incidence calculated as previously described.^18^ We defined the adjusted infection risk reduction (RR) as follows:

$$\text{RR}=\text{risk}{\text{ratio}}_{i,n}-1$$

where *i* is BNT162b2 or ChAdOx1 nCoV-19 vaccine, *n* is the number of days since vaccination (0–4, 5–11, 12–20, 21–44, 45–59), and the risk ratio is the coefficient of the vaccine status variables in the Poisson regression model.

We further tested the role of covariates in risk of infection after vaccination by running stratified Poisson models (adjusted for confounders). For this analysis, we considered all app responders who were vaccinated with BNT162b2 or ChAdOx1 nCoV-19 vaccines at least 12 days before having a test for SARS-CoV-2 positivity. Due to a relatively small sample size in some of the strata, we did not differentiate between vaccine types but pooled all vaccinated contributors.

Data were extracted and preprocessed using ExeTera version 0.3.2,^19^ a Python library developed at King's College London, and we did statistical analysis using Python version 3.7 (pandas, NumPy, and SciPy).

### Role of the funding source

ZOE Global developed the app for data collection as a not-for-profit endeavour. The funder had no role in study design, data analysis, data interpretation, or writing of the report.

## Results

Between Dec 8, 2020, and March 10, 2021, 655 590 vaccine doses were logged in the app in the UK, corresponding to 282 103 individuals (aged 16–99 years) who reported having received the first dose of the BNT162b2 vaccine, of whom 28 207 reported having had both doses of the BNT162b2 vaccine (a median of 41 days [IQR 21–63] apart), and 345 280 individuals who reported having had the first dose of ChAdOx1 nCoV-19. Participants joined the COVID Symptom Study app a mean of 288 days (SD 96) before being vaccinated. Users started logging adverse effects reports 0·79 days (SD 1·2) after they had the vaccine. 1 607 620 users were active in the app during the study period (logged at least an assessment since Dec 8), which represents 2·4% of the UK population. The mean age of app users was 50·6 years (SD 19·2), and 77 683 (4·8%) of them were health-care workers.

Users who reported receiving a BNT162b2 vaccine were slightly younger, had more comorbidities, and were more frequently female than users who reported receiving a ChAdOx1 nCoV-19 inoculation (table). Moreover, health-care workers were more likely to receive a BNT162b2 vaccine than ChAdOx1 nCoV-19 (appendix p 8). The age distribution of the included individuals is presented in the appendix (p 3).

**Table:** Demographic characteristics of the study population

		**BNT162b2**		**ChAdOx1 nCoV-19**
		First dose (N=282 103)	Second dose (N=28 207)	First dose (N=345 280)
Sex*				
	Female	173 866 (61·6%)	19 640 (69·6%)	199 269 (57·7%)
	Male	108 237 (38·4%)	8567 (30·4%)	146 011 (42·3%)
Age, years*†		62·0 (14·3); 64 (54–72)	61·0 (17·3); 59 (49–76)	63·3 (11·5); 65 (59–71)
Body-mass index, kg/m^2^‡		26·8 (5·5)	26·5 (5·3)	26·8 (5·3)
Health-care workers*		31 996 (11·3%)	8828 (31·3%)	9746 (2·8%)
Comorbidities*		77 433 (27·4%)	7617 (27·0%)	88 453 (25·6%)
Previous COVID-19*		14 369 (5·1%)	2251 (8·0%)	14 231 (4·1%)
Systemic side-effects				
	Any	38 155 (13·5%)	6216 (22·0%)	116 473 (33·7%)
	Headache	21 910 (7·8%)	3731 (13·2%)	78 734 (22·8%)
	Fatigue	23 674 (8·4%)	4064 (14·4%)	72 924 (21·1%)
	Chills and shiver	7166 (2·5%)	1812 (6·4%)	50 761 (14·7%)
	Diarrhoea	3885 (1·4%)	416 (1·5%)	7546 (2·2%)
	Fever	4236 (1·5%)	1076 (3·8%)	28 268 (8·2%)
	Arthralgia	9021 (3·2%)	1978 (7·0%)	39 648 (11·5%)
	Myalgia	6479 (2·3%)	1415 (5·0%)	24 274 (7·0%)
	Nausea	5926 (2·1%)	981 (3·5%)	19 509 (5·7%)
Local side-effects§				
	Any	150 023 (71·9%)	9025 (68·5%)	104 282 (58·7%)
	Pain	61 016 (29·2%)	4515 (34·3%)	33 939 (19·1%)
	Swelling	13 264 (6·4%)	1285 (9·8%)	9769 (5·5%)
	Tenderness	119 431 (57·2%)	6705 (50·9%)	87 609 (49·3%)
	Itch	6242 (3·0%)	840 (6·4%)	6934 (3·9%)
	Swollen armpit glands	2278 (1·1%)	549 (4·2%)	1994 (1·1%)
	Redness	7891 (3·8%)	953 (7·2%)	7431 (4·2%)
	Warmth	14 024 (6·7%)	1245 (9·4%)	14 033 (7·9%)
	Bruising	1872 (0·9%)	64 (0·5%)	4269 (2·4%)
Allergic reactions				
	Rash	682 (0·2%)	103 (0·4%)	1432 (0·4%)
	Skin burning	2075 (0·7%)	324 (1·1%)	5940 (1·7%)
	Red welts on face and lips	469 (0·2%)	59 (0·2%)	846 (0·2%)

Data are n (%), unless otherwise indicated.

* p<0·05 for the difference between the first dose of BNT162b2 and the first dose of ChAdOx1 nCoV-19 (Student's *t* test for continuous variables and χ^2^ test for categorical variables).

† Data are mean (SD); median (IQR).

‡ Data are mean (SD).

§ Denominators are 208 767 for the first BNT162b2 dose, 13 179 for the second BNT162b2 dose, and 177 655 for the first ChAdOx1 nCoV-19 dose.

**Figure 1:**
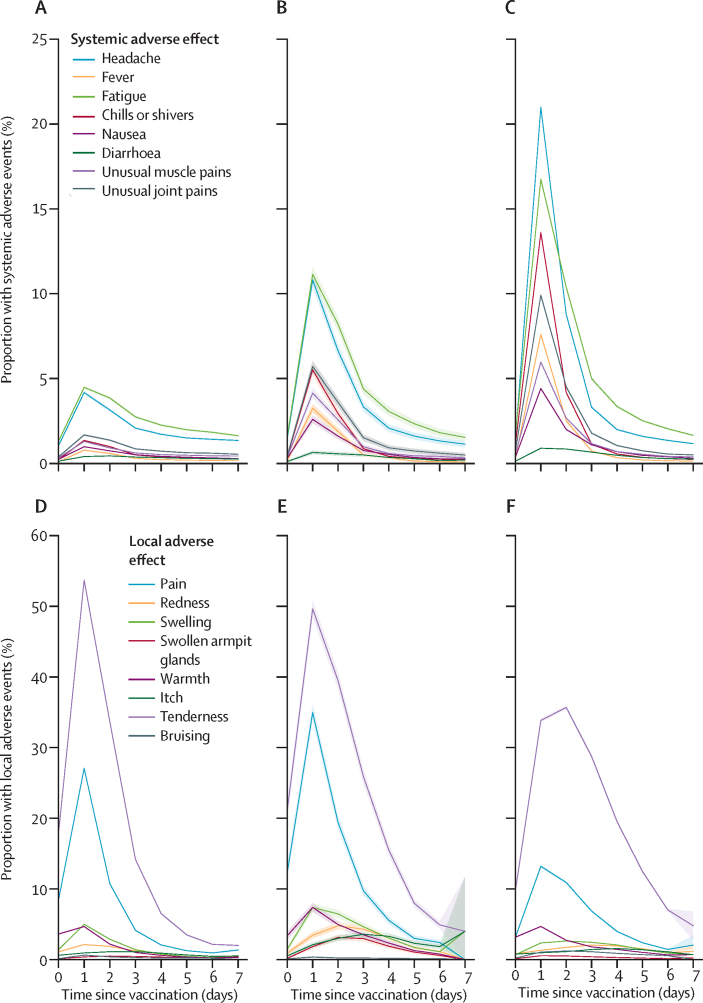
Proportion of participants self-reporting adverse effects to the COVID Symptom Study app within 8 days after vaccination The top row shows systemic effects and the bottom row shows local effects within 8 days after receipt of the first dose (A, D) or second dose (B, E) of the BNT162b2 vaccine or the first dose of the ChAdOx1 nCoV-19 vaccine (C, F). Shading indicates 95% CIs.

Among vaccinated app users, 159 101 (25·4%) of 627 383 indicated having one or more systemic adverse effect, and 257 209 (66·2%) of 388 430 reported one or more local adverse effect (table). The most commonly reported systemic side-effects were fatigue and headache overall (table) and by strata (appendix p 9). These were most frequently reported within the first 24 h after vaccination and lasted a mean of 1·01 days (SD 0·1; figure 1). Tenderness and local pain around the injection site were the most frequently reported local effects (table), occurring most often on the day after injection and lasting a mean of 1·02 days (SD 0·15; figure 1). Other side-effects, including allergic skin reactions such as skin burning, rashes, and red welts on the lips and face, were reported by 10 860 (1·7%) of 627 383 users across both types of vaccine (table; appendix p 9). In an exploratory analysis, we assessed the association between symptom reporting and socioeconomic status measured as index of multiple deprivation,^20^ and we found a modest association (*r*=0·021 [95% CI 0·019–0·025]), corresponding to 0·04% of the variance in symptoms reporting.

**Figure 2:**
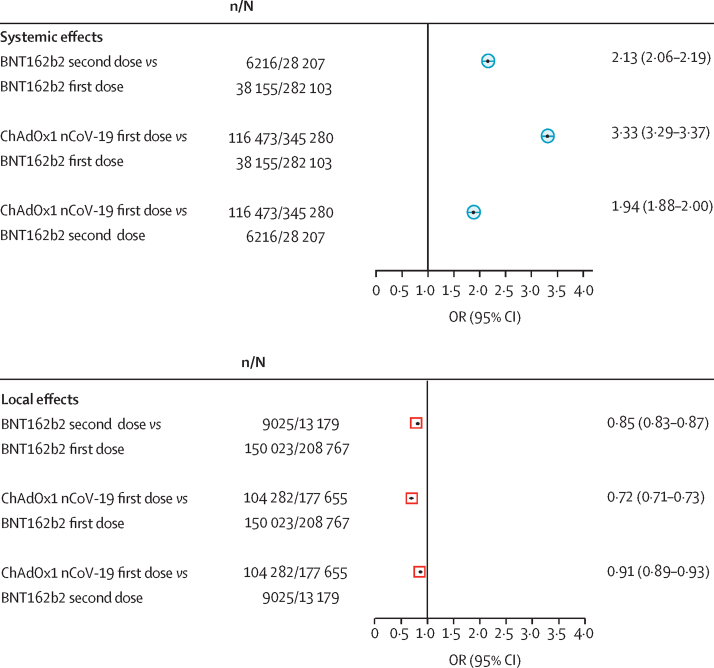
Comparison of adverse effects self-reported to the COVID Symptom Study app between vaccine types and doses ORs for comparisons of the first or second doses of BNT162b2 versus the first dose of ChAdOx1 nCoV-19 were adjusted using Pearl's back-door method.^16^ OR=odds ratio.

In the 28 207 individuals who reported having two BNT162b2 doses, 3325 (11·7%) reported at least one systemic effect after the first dose compared with 6216 (22·0%) after the second dose (p<0·0001; figure 2). When comparing systemic effects after one dose of each vaccine, reactogenicity was significantly higher in individuals who had one dose of the ChAdOx1 nCoV-19 vaccine than in those who had one dose of the BNT162b2 vaccine (116 473 [33·7%] of 345 280 compared with 38 155 [13·5%] of 282 103; adjusted p<0·0001).

By contrast, local effects were less commonly reported after the second dose than after the first dose of BNT162b2 (9025 [68·5%] of 13 179 *vs* 150 023 [71·9%] of 208 767; p<0·0001). Moreover, local effects were less commonly reported after the first ChAdOx1 nCoV-19 injection (104 282 [58·7%] of 177 655) than after the first BNT162b2 injection (adjusted p<0·0001; figure 2).

When comparing the second BNT162b2 dose with the first ChAdOx1 nCoV-19 dose, we found that systemic effects occurred more frequently after the first ChAdOx1 nCoV-19 dose than after the second BNT162b2 dose (adjusted p<0·0001; figure 2), whereas local effects were less likely to appear after the first ChAdOx1 nCoV-19 injection than after the second BNT162b2 dose (adjusted p<0·0001; figure 2).

**Figure 3:**
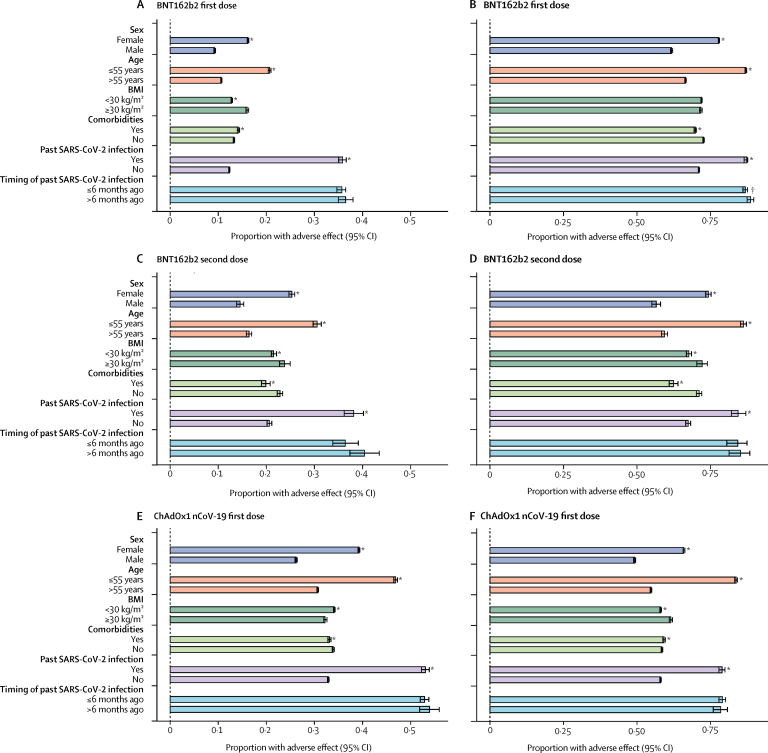
Adverse effects self-reported to the COVID Symptom Study app after COVID-19 vaccination, stratified by sex, age, BMI, health status, and previous SARS-CoV-2 test status Proportions with systemic adverse effects (A, C, E) and local adverse effects (B, D, F) are shown. Error bars represent 95% CIs. Numbers in each strata are reported in the appendix (pp 13–14). BMI=body-mass index. *p<0·01. †p<0·05.

We then tested whether adverse effects varied across individuals' characteristics, such as age and BMI groups, sex, and health status. The proportion of participants who reported at least one systemic effect after the first dose was significantly higher among people aged 55 years or younger than among those older than 55 years for both vaccines. After the first dose of BNT162b2, 16 733 (20·7%) of 80 879 people aged 55 years or younger reported at least one systemic effect compared with 21 422 (10·6%) of 201 224 people older than 55 years (OR 2·19 [95% CI 2·14–2·24]; p<0·0001). For ChAdOx1 nCoV-19, 30 487 (46·9%) of 65 034 people aged 55 years or younger reported at least one systemic effect after the first dose compared with 85 986 (30·7%) of 280 243 older than 55 years (1·99 [1·96–2·03]; p<0·0001). Women were more likely to report adverse effects than men (28 140 [16·2%] of 173 866 *vs* 10 015 [9·3%] of 108 237 after the first dose of BNT162b2, OR 1·89 [95% CI 1·85–1·94], p<0·0001; 78 222 [39·3%] of 199 269 *vs* 38 251 [26·2%] of 146 011 after the first dose of ChAdOx1 nCoV-19, 1·82 [1·79–1·85], p<0·0001). Although there were some differences between strata of BMI and co-morbidities, there was no clear trend across vaccines and doses (figure 3).

We observed the same trend for local effects as for systemic effects, whereby app users aged 55 years or younger reported more local side-effects than participants older than 55 years (figure 3), and women were more likely to report local side-effects than men (figure 3; appendix p 11). Similar levels of side-effects were seen regardless of the levels of completeness of reporting by app users (appendix p 12).

Given preliminary evidence from small studies^21^, ^22^, ^23^ suggesting that reactogenicity is higher among individuals previously infected with SARS-CoV-2, we investigated the extent to which previous SARS-CoV-2 infection (based on self-reported previous positive PCR or lateral flow result) was associated with reports of adverse effects. Individuals vaccinated with a single dose of BNT162b2 were more likely to report systemic effects if they had a previous SARS-CoV-2 positive test than were those without known past infection (5148 [35·8%] of 14 369 *vs* 33 007 [12·3%] of 267 734; OR 3·97 [95% CI 3·83–4·12], p<0·0001). A similar effect was seen for ChAdOx1 nCoV-19 first dose inoculation (7551 [53·1%] of 14 231 with past infection *vs* 108 922 [32·9%] of 331 049 without past infection; 2·31 [2·23–2·38], p<0·0001; figure 3) and BNT162b2 second dose inoculation (859 [38·2%] of 2251 *vs* 5357 [20·6%] of 25 956; 2·37 [2·17–2·60], p<0·0001). Local effects were similarly higher in individuals previously infected than in those without known past infection for both vaccines (figure 3). No consistent difference in occurrence of systemic or local adverse effects was observed between individuals who reported a positive test result within the past 6 months and those who reported they received a positive test result at least 6 months ago (figure 3).

**Figure 4:**
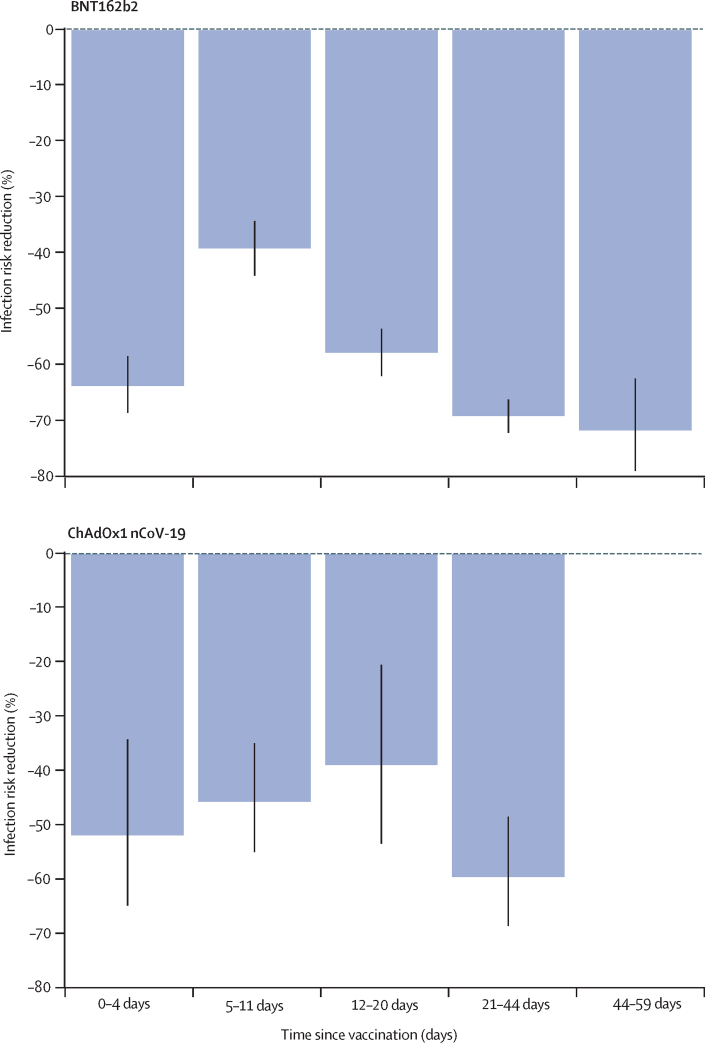
Infection risk reduction after the first dose in app users who have been vaccinated and subsequently tested, as a function of days since vac The bar chart represents the risk reduction for infection of the vaccinated groups (those who logged at least one PCR or lateral flow test result after vaccination) compared with the unvaccinated group, by vaccine type and days since vaccination. The black lines show 95% CIs.

We also investigated infection rates after the first vaccine dose in a subset of 67 293 app users who received BNT162b2 and 36 329 who received ChAdOx1 nCoV-19 in the study period and logged at least one PCR or lateral flow test after vaccination. We compared the test results of this population with those of 464 356 unvaccinated app users who had a PCR or lateral flow test result between Jan 4, and March 10, 2021 (appendix pp 4, 8). 3106 of 103 622 vaccinated individuals and 50 340 of 464 356 unvaccinated controls tested positive for SARS-CoV-2 infection. As UK guidelines stipulate that individuals need to be free of symptoms to be vaccinated, we found vaccinated participants to have a lower infection risk at the time of the vaccination than unvaccinated participants (RR for BNT162b2 −64% [95% CI −69 to −59]; RR for ChAdOx1 nCoV-19 −52% [95% CI −65 to −34]). We observed that 5–11 days after vaccination, the infection rates in the vaccinated group were only slightly below those of the unvaccinated group (figure 4), whereas 12–20 days after vaccination, infection risk in the vaccinated group was significantly lower than in the unvaccinated group (RR for BNT162b2 −58% [95% CI −62 to −54]; RR for ChAdOx1 nCoV-19 −39% [95% CI −53 to −21]), after adjusting for population differences in the vaccinated groups using Poisson regressions. We observed a further reduction in infection risk after one dose of the BNT162b2 vaccine when compared with unvaccinated controls at 21–44 days after vaccination (RR −69% [95% CI −72 to −66]) and at 45–59 days after vaccination (−72% [–79 to −63]; figure 4). The RR after one dose of ChAdOx1 nCoV-19 compared with unvaccinated controls was −60% (95% CI −68 to −49) at 21–44 days after vaccination.

Finally, we tested the role of covariates in risk of infection after vaccination. We observed a larger risk reduction in vaccinated participants aged 55 years or younger (RR −70% [95% CI −72 to −68) than in those older than 55 years (−61% [–64 to −57]). Similarly, individuals without comorbidities had a larger risk reduction (−69% [–71 to −68]) than those with at least one comorbidity (−54% [–59 to −48]). Borderline differences were observed for BMI (RR for BMI <30 kg/m^2^ −69% [95% CI −71 to −67]; RR for BMI ≥30 kg/m^2^ −63% [−67 to −59]) and sex (RR for female −69% [95% CI −71 to −67]; RR for male −61% [–66 to −57]; appendix p 5).

## Discussion

In this large-scale, community-based study in the UK, we have investigated adverse effects and infection rates following administration of the two COVID-19 vaccines that are in use in the UK. The overall mean age of the vaccinated app users was higher than that of the general population (40·3 years)^24^ yet was lower than those of the samples within other UK COVID-19 effectiveness studies,^11^, ^12^ largely because of the presence of a small proportion of health-care workers among the participants of this study. However, our study population was considerably older than the study populations of the phase 3 trials.^3^, ^6^, ^7^ We found that systemic adverse effects, including headache and fatigue, affected fewer than one in four people and were less common in the community than expected from clinical trials. For example, in phase 3 clinical trials of the BNT162b2 vaccine,^3^ the most common events after the first dose were injection-site pain (71–83%), fatigue (34–47%), and headache (25–42%). However, in our community analysis, less than 30% of users complained of injection-site pain and less than 25% of fatigue and headache after the first dose. Although side-effects were significantly more prevalent in women than in men, in people aged 55 years or younger than in those older than 55 years, and after the second than after the first dose, they occurred at much lower frequencies than expected from the published literature. For instance, whereas 51–59% of participants reported fatigue after the second BNT162b2 dose in the phase 3 trial of that vaccine,^3^ fatigue was reported by less than 15% of participants after the second dose in our study. Additionally, our data provide evidence from the community to support early reports of higher frequency of side-effects in younger than in older individuals.^3^, ^7^

Similarly, rates of side-effects following the ChAdOx1 nCoV-19 vaccine were lower than expected.^7^ The phase 2–3 trial of the ChAdOx1 nCoV-19 vaccine^7^ reported systemic adverse effects in 88% of participants aged 18–55 years who received the first injection, whereas we found a lower rate of 33·7% after the first dose in the overall sample and 46·9% in individuals aged 18–55 years (data not shown). Individuals vaccinated with the ChAdOx1 nCoV-19 vaccine were more likely to experience systemic side-effects than those who had been given the BNT162b2 vaccine, but in our study 89% of respondents who logged at least one systemic effect after the ChAdOx1 nCoV-19 vaccine did not report any systemic effects after 3 days, and 98·3% did not report any after 1 week.

Individuals with evidence of past SARS-CoV-2 infection were also more likely to have adverse effects than those without evidence of past infection with both vaccines. It is possible, although it remains to be tested, that this increased reactogenicity relates to increased immunogenicity. It has been shown that vaccines have increased immunogenicity in individuals with past infection and these people have higher antibody titres than those without previous infection.^22^, ^23^, ^25^

We observed an infection risk reduction at 21–44 days after vaccination in all vaccinated users compared with unvaccinated controls (RR was −69% [95% CI −72 to −66] for BNT162b2 and −60% [–68 to −49] for ChAdOx1 nCoV-19). The reduction of infection was lower in individuals older than 55 years than those aged 55 years or younger, in those with one or more comorbidities than in those without comorbidities, and in individuals with a BMI of 30 kg/m^2^ or higher than in those with a BMI of less than 30 kg/m^2^ (appendix p 5). A preprint based on data from Israel suggested that a single dose of BNT162b2 might not provide enough protection;^26^ however, a re-analysis of the same dataset indicated that after 14 days the effectiveness of a single dose of vaccine was about 90%.^5^ Although our data, due to their observational nature, does not allow us to comment directly on effectiveness, the observed decrease of infection over time seems to be in line with efficacy reported in the BNT162b2 phase 3 trial^3^ and supports the UK Government's decision to delay the timing of the second injection to 12 weeks to maximise the number of people receiving at least one dose. Long-term surveillance for SARS-CoV-2 protection in individuals who have received delayed second doses of BNT162b2 compared with those receiving second doses according to initial guidelines (ie, 21 days after the first dose) will be required to determine whether these initial protection estimates persist.

Strengths of our study include its large sample size; capture of data on SARS-CoV-2 RT-PCR or lateral flow test results, regardless of symptoms; the prospective real-time capture of information on symptoms; and the availability of both BNT162b2 and ChAdOx1 nCoV-19 vaccines in the UK, which allowed cross-vaccine comparison. Our study also has several limitations. We used self-reported data, which can introduce information bias, including misclassification, or effect bias exposure. Also, some participants might be more likely to report symptoms than others, and there is the potential for users to drop out of reporting in the app. Participants using the app were a self-selected group and not representative of the general population, as has been observed in other digital platform studies.^27^ Users of a participatory platform (as well as participants in all voluntary studies, including clinical trials) are likely to be more interested in health, and might behave differently to the general population as a result. Previous work has shown that data from our app is able to produce estimates of population-level disease prevalence that agree well with surveys with random, representative designs,^18^, ^28^ suggesting that behavioural issues are not substantially biasing our app population. As with other studies examining COVID-19 vaccine effects in the general community, our data are limited by the vaccine rollout's focus on health-care workers, elderly people, and people who are clinically vulnerable.^2^ Moreover, our results might have been affected by collider bias (ie, when a risk factor and an outcome both affect the likelihood of being sampled)^29^ if both vaccination status and COVID positivity influenced the probability of participation in the app. However, given that strong reductions in COVID hospitalisations after vaccination were observed in nationwide studies in Scotland^12^ and England,^11^ we believe that collider bias is unlikely to underlie the reduction in infections seen in our data. Recipients of the ChAdOx1 nCoV-19 vaccine might differ from recipients of the BNT162b2 vaccine by age or dependency. Although we adjusted for population differences across the BNT162b2, ChAdOx1 nCoV-19, and unvaccinated control groups, our estimates of infection rates after vaccination might not have fully adjusted for case-mix and therefore are preliminary. Furthermore, because the ChAdOx1 nCoV-19 vaccine started being rolled out in January, 2021, and the second dose is to be administered at 12 weeks, no app users had received two doses of ChAdOx1 nCoV-19 at the time of this report. The completeness of reporting was higher for systemic effects than for local effects, which might have introduced some bias (appendix p 12). Some severe side-effects might have been missed if app users experiencing them were unable to use the app to report side-effects. However, we saw substantially lower rates of severe and mild side-effects than observed in phase 3 trials, making the missing of severe side-effects an unlikely explanation for the lower prevalence of side-effects seen in our data. Furthermore, we cannot rule out the presence of selection bias in who was tested after vaccination, as we know that health-care workers are tested more frequently than people in the general population, even if they are asymptomatic. This is an observational study, with data captured during a specific timeframe, and our study design does not allow an inference of causality. Also, we evaluated only short-term adverse effects, and long-term surveillance in the general population will be required to investigate possible future effects. Finally, the systemic side-effects were collected from daily reports within 1 week from the injection date, so we cannot rule out that these effects might not be vaccine related. We also had insufficient power to assess differential rates by ethnic group.

In conclusion, short-term adverse effects of both vaccines are moderate in frequency, mild in severity, and short-lived. Adverse effects are more frequently reported in younger individuals, women, and among those who previously had COVID-19. The post-vaccine symptoms (both systemic and local) often last 1–2 days from the injection. Our data could be used to inform people on the likelihood of side-effects on the basis of their age and sex and the type of vaccine being administered. Furthermore, our data support results from randomised controlled trials in a large community-based scenario showing evidence of reduction in infection after 12 days and substantial protection after 3 weeks.

## Data sharing

Anonymised research data are shared with third parties via Health Data Research UK (HDRUK.ac.uk). US investigators are encouraged to coordinate data requests through the Coronavirus Pandemic Epidemiology (COPE) consortium (www.monganinstitute.org/cope-consortium). Data updates can be found at https://covid.joinzoe.com.

## Declaration of interests

CM reports grants from Chronic Disease Research Foundation (CDRF) during the conduct of the study. JW, AM, LP, CH, SS, and JC report being employees of ZOE Global during the conduct of the study. ATC reports grants from Massachusetts Consortium on Pathogen Readiness during the conduct of the study, and personal fees from Bayer Pharma, Pfizer, and Boehringer Ingelheim, outside the submitted work. DAD reports grants from National Institutes of Health (NIH), Massachusetts Consortium on Pathogen Readiness, and American Gastroenterological Association, during the conduct of the study, and that he served as a co-investigator on an unrelated nutrition trial sponsored by ZOE Global. CHS reports grants from Alzheimer's Society during the conduct of the study. AMV reports grants from Medical Research Council (MRC) and personal fees from ZOE Global, during the conduct of the study. ALG reports having shares in AstraZeneca and receiving grants from Novavax, outside the submitted work. CJS reports grants from CDRF, MRC, and Wellcome Trust, during the conduct of the study. SO reports grants from Wellcome Trust, UK Research and Innovation (UKRI), and CDRF, during the conduct of the study. TDS reports being a consultant for ZOE Global, during the conduct of the study. All other authors declare no competing interests.
