# Supplementary material for: Vaccine side-effects and SARS-CoV-2 infection after vaccination in users of the COVID Symptom Study app in the UK: a prospective observational study
Source: Lancet Infect Dis. 2021 Jul;21(7):939–49. doi: 10.1016/S1473-3099(21)00224-3 (PMC8078878; doi:10.1016/S1473-3099(21)00224-3)
Supplement: Supplementary appendix [file mmc1.pdf]

THE LANCET
Infectious Diseases

Supplementary appendix

This appendix formed part of the original submission and has been peer reviewed. We post it as supplied by the authors.

Supplement to: Menni C, Klaser K, May A, et al. Vaccine side-effects and SARS-CoV-2 infection after vaccination in users of the COVID Symptom Study app in the UK: a prospective observational study. *Lancet Infect Dis* 2021; published online April 27. https://doi.org/10.1016/S1473-3099(21)00224-3.

Vaccine after effects and post-vaccine infection in a community setting - results from the COVID Symptom Study app

Cristina Menni, PhD, Kerstin Klaser, MSc, Anna May, MA, Lorenzo Polidori, MSc, Joan Capdevila, PhD, Panayiotis Louca, MSc, Carole H. Sudre, PhD, Long H. Nguyen, MD, David A. Drew, PhD, Jordi Merino, PhD, Christina Hu, BA, Somesh Selvachandran, MEng, Michela Antonelli, PhD, Benjamin Murray, MSc, Liane S. Canas, PhD, Erika Molteni, PhD, Mark S. Graham, PhD, Marc Modat, PhD, Amit D. Joshi, PhD, Massimo Mangino, PhD, Prof Alexander Hammers, PhD, Anna L Goodman, DPhil, Prof Andrew T. Chan, MD, Jonathan Wolf, MA, Claire J. Steves, PhD, Prof Ana M. Valdes, PhD, Prof Sebastien Ourselin, PhD, Prof Tim D. Spector, MD

APPENDIX

Table of Contents

Supplementary Figure 1. Consort diagram 2

Supplementary Figure 2. Age distribution of the study population. 3

Supplementary Table 1. Questions from the ZOE COVID Symptom Study app 6

Supplementary Table 2. Demographic characteristics of the vaccinated then subsequently tested study population, compared to the unvaccinated control group. 8

Supplementary Table 3. Prevalence of side effects by demographic strata and by dose and type of vaccine (unadjusted % are presented). 9

Supplementary Table 4. Demographic and other characteristics of participants who reported side effects and of those not reporting them. 11

Supplementary Table 5. Characteristics of app users reporting side effects by completeness of reporting. 12

Supplementary Table 6. Proportion of individuals reporting systemic side-effects after BNT162b2 first dose, BNT162b2 second dose or ChAdOx1 nCoV-19 first dose by strata. 13

Supplementary Table 7. Proportion of individuals reporting local side-effects after BNT162b2 first dose, BNT162b2 second dose or ChAdOx1 nCoV-19 first dose by strata. 14

Supplementary Text 15

Supplementary Figure 1. Consort diagram

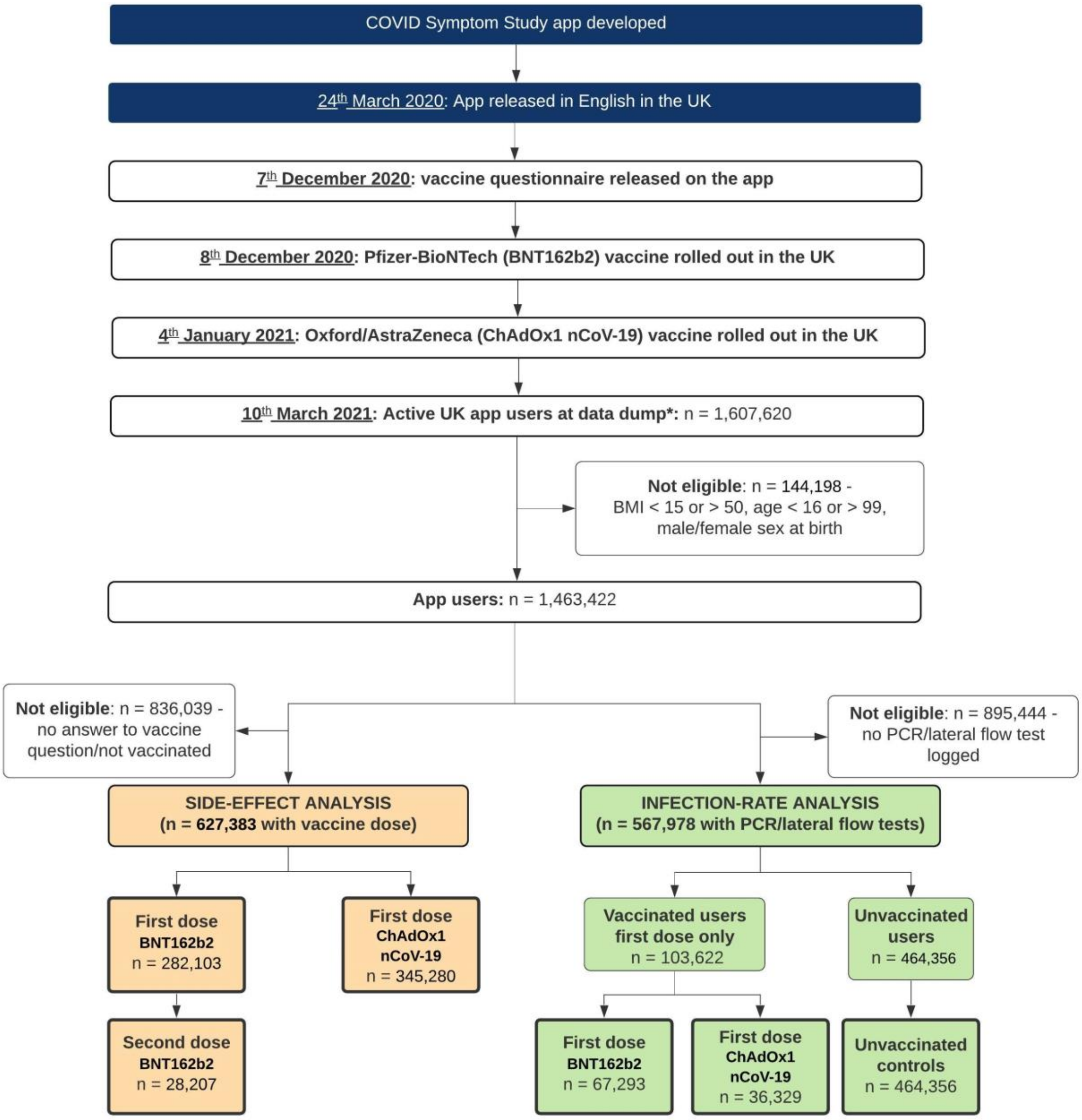

Supplementary Figure 2. Age distribution of the study population.

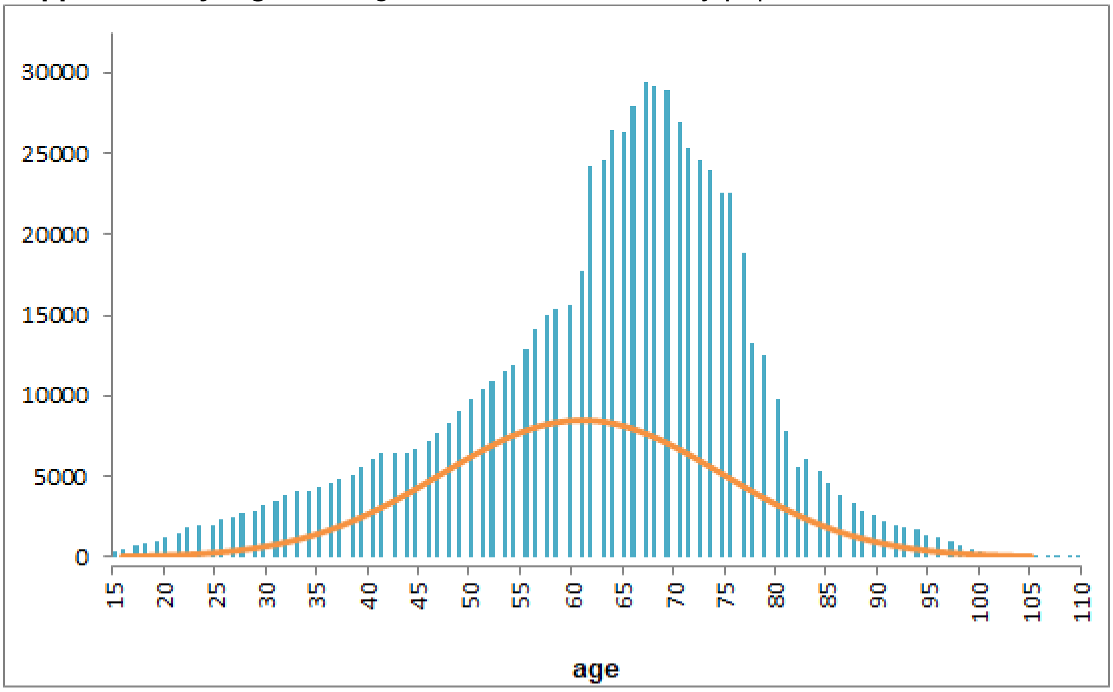

Supplementary Figure 3. Kaplan-Meier plot showing the probability of remaining free of SARS-CoV-2 infection over time for individuals who had received the first dose of the BNT162b2 vaccine or the ChAdOx1 vaccine. Unvaccinated individuals are selected from among all unvaccinated users to have had a PCR/lateral flow test on the same day as a vaccinated app user and to have their same age, sex, comorbidity, obesity. Estimates, sample sizes and statistical significance are those from Figure 4.

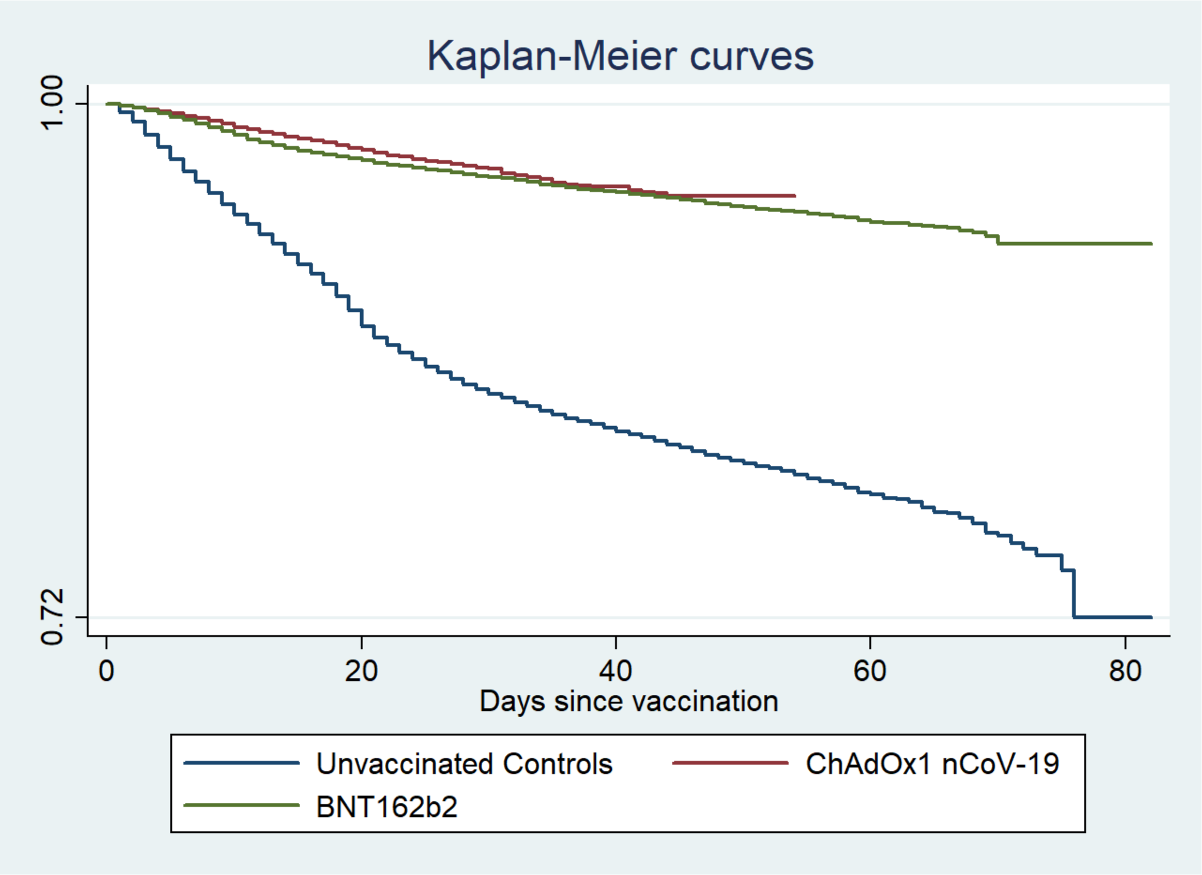

Supplementary Figure 4. Stratified infection risk reduction after the first dose in users who have been vaccinated and then subsequently tested. The bar chart represents the ≥12 days risk reduction of those vaccinated with BNT162b2 or ChAdOx1 nCoV-19 compared to the unvaccinated group, for each of the strata. The black lines represent 95%CIs. The strata are as follows: age: ≤55 and >50 year, obesity: BMI <30 and ≥30 kg/m^2, comorbidities: no comorbidities or has at least one comorbidity (diabetes, heart, lung or kidney disease, had chemotherapy, had cancer, takes immunosuppressants), sex: female or male. N: vaccinated= 103,622, control = 464,356.

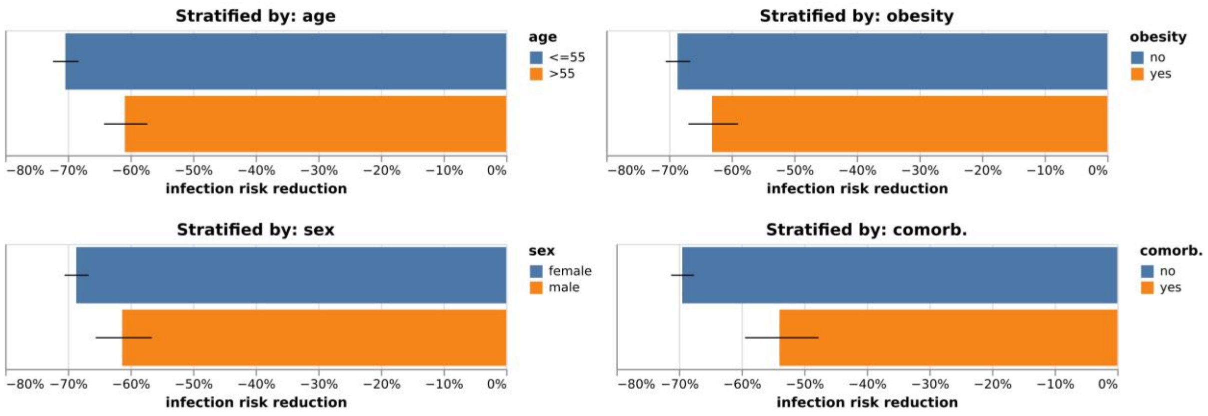

Supplementary Table 1. Questions from the ZOE COVID Symptom Study app

App Screen Name	App Display Text	Data Value
**About your first dose**	**What is the name of the vaccine?**	*Astrazeneca*<br>*Pfizer*<br>*Moderna*<br>*Unsure*
	**When was your injection?**	*Insert date*
**About your second dose**	**What is the name of the vaccine?**	*Astrazeneca*<br>*Pfizer*<br>*Moderna*<br>*Unsure*
	**When was your injection?**	*Insert date*
**Report local effects**	**Check all that apply:**	*Pain*<br>*Redness*<br>*Swelling*<br>*Swollen glands in the armpit*<br>*Warmth*<br>*Itch*<br>*Tenderness*<br>*Bruising*<br>*Other*
**How you feel**	**How do you feel physically right now?**	*I feel physically normal*<br>*I'm not feeling quite right*
**Describe symptoms**	**Check all that apply**<br><br>*(only those that we consider systemic or allergic effects are listed)*	*Fever*<br><br>*Chills or shivers*<br><br>*Unusual fatigue (mild, severe)*<br><br>*Rash on your arm or torso*<br><br>*Raised, red, itchy welts on the skin or sudden swelling of the face or lips*<br><br>*Strange, unpleasant sensations in your skin like pins and needles or burning*<br><br>*Unusual strong muscle*

		pains or aches<br><br>*Unusual joint pains or aches*<br><br>*Headache*<br><br>*Nausea or vomiting*<br><br>*Diarrhoea*

Supplementary Table 2. Demographic characteristics of the vaccinated then subsequently tested study population, compared to the unvaccinated control group.

	**BNT162b2**	**ChAdOx1 nCoV-19**	**Unvaccinated Controls**
N	67293	36329	464356
Positive PCR tests	2465	641	50340
Females, n (%)	49304 (73.3%) *	24054 (66.2%) *	299412 (64.5%)
Age, years mean(SD)	54.5 (14.3) *	60.8 (13.5) *	49.4 (14.6)
BMI, Kg/m2 mean(SD)	26.8 (5.6) *	26.7 (5.3) *	26.6 (5.5)
HCWs, n (%)	20421 (30.3%) *	3817 (10.5%) *	32454 (7%)
Comorbidities, n (%)	14411 (21.4%) *	9638 (26.5%) *	79753 (17.2%)

* ($P<0.05$) between BNT162b2 dose 1 and unvaccinated controls and ChAdOx1 nCoV-19 dose 1 and unvaccinated controls (two sample t-test for continuous variables and chi-squared test for categorical variables)

Supplementary Table 3. Prevalence of side effects by demographic strata and by dose and type of vaccine (unadjusted % are presented).

Group	All vaccinated	BNT162b2 first dose	BNT162b2 second dose	ChAdOx1 nCoV-19 first dose
**Age <= 55**	Headache (23.99%), fatigue (22.19%), Chills or shivers (13.75%)	Headache (13.23%) Fatigue (12.88%) Unusual joint pains (5.22%)	Headache (20.33%) Fatigue (19.80%) Unusual Joint Pain (10.36%)	Headache (34.70%) Fatigue (31.14%) Chills or shivers (24.26%)
**Age > 55**	Headache (14.24%), fatigue (14.0%), chills or shivers (8.20%)	Fatigue (6.59%) Headache (5.57%) Unusual joint pains (2.38%)	Fatigue (10.84%) Headache (8.52%) Unusual joint pains (4.80%)	Headache (20.04%) Fatigue (18.79%) Chills or shivers (12.48%)
**BMI >= 30**	Headache (16.74%), fatigue (16.41%), chills or shivers (8.84%)	Fatigue (10.13%) , Headache (9.70%) , Unusual joint pains (4.46%)	Fatigue (15.51%) Headache (14.87%) Unusual joint pains (9.19%)	Headache (21.73%) Fatigue (20.77%) Chills or shivers (13.02%)
**BMI < 30**	Headache (16.44%), fatigue (15.76%), chills or shivers (9.67%)	Fatigue (7.91%) Headache (7.23%) Unusual joint pains (2.84%)	Fatigue (14.12%) Headache (12.81%) Unusual Joint Pains (6.46%)	Headache (23.09%) Fatigue (21.21%) Chills or shivers (15.15%)
**Not an HCW**	Headache (15.11%), fatigue (14.91%), chills or shivers (8.90%)	Fatigue (7.23%), Headache (6.12%) , Unusual joint pains (2.61%)	Fatigue (9.01%) Headache (6.31%) Unusual Joint Pains (3.61%)	Headache (21.07%) Fatigue (19.88%) Chills or shivers (13.49%)
**HCW**	Headache (19.19%), Fatigue (17.99%), Chills or shivers (9.58%)	Headache (11.7%), Fatigue (10.94%), Unusual joint pains (5.0%)	Fatigue (17.61%) Headache (17.21%) Unusual Joint Pains (9.40%)	Headache (31.92%) Fatigue (28.97%) Chills or shivers (21.63%)
**Has Comorbidity**	Fatigue (16.14%) Headache (16.01%) Chills or shivers (8.90%)	Fatigue (9.08%) Headache (7.95%)	Fatigue (13.36%) Headache (11.59%)	Headache (22.31%) Fatigue (21.46%) Chills or

		Unusual Joint Pains (3.48%)	Unusual Joint Pains (6.46%)	shivers (13.96%)
**Does not Have Comorbidity**	Headache (16.69%)<br>Fatigue (15.81%)<br>Chills or shivers (9.71%)	Fatigue (8.13%)<br>Headache (7.69%)<br>Unusual Joint Pains (3.09%)	Fatigue (14.79%)<br>Headache (13.83%)<br>Unusual Joint Pains (7.22%)	Headache (22.97%)<br>Fatigue (21.00%)<br>Chills or shivers (14.96%)

Supplementary Table 4. Demographic and other characteristics of participants who reported side effects and of those not reporting them.

	Reported local adverse effects?		Report systemic adverse effects?	
	No	***Yes***	***No***	***Yes***
N	130,633	255,789	468,282	159,101
Age, mean (SD)	70.49 (10.63)*	62.71(13.93)	63.79(12.50)*	59.50(13.25)
BMI, mean (SD)	26.45 (4.86)	26.46(5.20)	26.82 (5.31)*	26.73(5.66)
Female %	47.70%*	66.16%	56.22%*	69.06%
HCW %	6.05%*	17.81%	9.10%*	11.01%
Comorbidities %	29.10%*	27.65%	26.69%*	26.08%

*significantly (P<0.05) different between those who reported adverse effects and those who didn’t (two sample t-test for continuous variables and chi-squared test for categorical

Supplementary Table 5. Characteristics of app users reporting side effects by completeness of reporting.

	**Doses with local adverse effects reports for full week after vaccine?**		**Doses with systemic adverse effects reports for full week after vaccine?**	
	No	***Yes***	***No***	***Yes***
N	316,164	83,437	455,334	200,256
Age, mean(SD)	64.97 (13.74)	66.61 (12.34)	61.3 (13.33)	65.8 (11.04)
BMI, mean(SD)	26.46 (5.10)	26.42 (5.05)	26.9 (5.50)	26.5 (5.14)
Female %	60.02%	59.75 %	60.3%	57.71%

Supplementary Table 6. Proportion of individuals reporting systemic side-effects after BNT162b2 first dose, BNT162b2 second dose or ChAdOx1 nCoV-19 first dose by strata.

	**BNT162b2 first dose (*n = 282,103*)**			**BNT162b2 second dose (*n = 28,207*)**			**ChAdOx1 nCoV-19 first dose (*n = 345,280*)**		
**Report systemic side-effects**	n	N	%	n	N	%	n	N	%
**Age**									
≤ 55	16,733	80,879	20.7	3,434	11,236	30.6	30,487	65,037	46.9
> 55	21,422	201,224	10.6	2,782	16,971	16.4	85,986	280,243	30.7
**Sex**									
Female	28,140	173,866	16.2	4,969	19,640	25.3	78,222	199,269	39.3
Male	10,015	108,237	9.3	1,247	8,567	14.6	38,251	146,011	26.2
**BMI**									
< 30	28,374	221,114	12.8	4,849	22,471	21.6	92,802	271,938	34.1
≥ 30	9,781	60,989	16.0	1,367	5,736	23.8	23,671	73,342	32.3
**Comorbidities**									
Yes	10,991	77,433	14.2	1,515	7,617	19.9	29,311	88,453	33.1
No	27,164	204,670	13.3	4,701	20,590	22.8	87,162	256,827	33.9
**Prior COVID**									
Yes	5,148	14,369	35.8	859	2,251	38.2	7,551	14,231	53.1
No	33,007	267,734	12.3	5,357	25,956	20.6	108,922	331,049	32.9
**Time prior COVID**									
≤ 6 months	3,726	10,472	35.6	460	1,263	36.4	6,333	11,972	52.9
> 6 months	1,422	3,897	36.5	399	988	40.4	1,218	2,259	53.9

n= number of people reporting side effects in a stratum; N=Total number of people in the stratum

Supplementary Table 7. Proportion of individuals reporting local side-effects after BNT162b2 first dose, BNT162b2 second dose or ChAdOx1 nCoV-19 first dose by strata.

	**BNT162b2 first dose (*n = 208,767*)**			**BNT162b2 second dose (*n = 13,179*)**			**ChAdOx1 nCoV-19 first dose (*n = 177,655*)**		
**Report local side-effects**	n	N	%	n	N	%	n	N	%
**Age**									
<55	47,707	54,830	87.0	3,866	4,485	86.2	20,348	24,313	83.7
>55	102,316	153,937	66.5	5,159	8,694	59.3	83,934	153,342	54.7
**Sex**									
Female	101,958	130,963	77.9	6,593	8,877	74.3	66,320	100,515	66.0
Male	48,065	77,804	61.8	2,432	4,302	56.5	37,962	77,140	49.2
**BMI**									
< 30	121,107	168,430	71.9	7,294	10,780	67.7	83,445	143,766	58.0
> 30	28,916	40,337	71.7	1,731	2,399	72.2	20,837	33,889	61.5
**Comorbidities**									
Yes	38,718	55,500	69.8	2,490	3,988	62.4	31,520	53,228	59.2
No	111,305	153,267	72.6	6,535	9,191	71.1	72,762	124,427	58.5
**Prior COVID**									
Yes	9,086	10,405	87.3	697	823	84.7	4,814	6,101	78.9
No	140,937	198,362	71.1	8,328	12,356	67.4	99,468	171,554	58.0
**Time prior COVID**									
< 6 months	6,432	7,410	86.8	364	432	84.3	3,945	4,993	79.0
> 6 months	2,654	2,995	88.6	333	391	85.2	869	1,108	78.4

n= number of people reporting side effects in a stratum; N=Total number of people in the stratum

Supplementary Text

$$OR = \frac{\dfrac{P\left(R|V_{m,n}\right)}{1 - P\left(R|V_{m,n}\right)}}{\dfrac{P\left(R|V_{k,j}\right)}{(1 - P(R|V_{k,j})}}$$

where *m, k* is [ChAdOx1 nCoV-19 OR BNT162b2] and *n,j* is [first dose OR second dose], R is adverse effects, $V_{m.n}$ and $V_{k,j}$ indicate the vaccine doses we are comparing.

So $P(R|V_{k,j})$ is the Pearl's back-door adjusted probability of having adverse effects (local or systemic) after the *j*-th dose of the *k*-th vaccine type.
